# A national study of chaplaincy services and end-of-life outcomes

**DOI:** 10.1186/1472-684X-11-10

**Published:** 2012-07-02

**Authors:** Kevin J Flannelly, Linda L Emanuel, George F Handzo, Kathleen Galek, Nava R Silton, Melissa Carlson

**Affiliations:** 1 The Spears Research Institute, HealthCare Chaplaincy, 307 East 60th Street, New York, NY, 10022, USA; 2 Buehler Center on Aging, Health & Society, Northwestern University Feinberg School of Medicine, 750 N. Lake Shore Drive, Suite 601, Chicago, IL, 60611, USA; 3 HealthCare Chaplaincy, 307 East 60th Street, New York, NY, 10022, USA; 4 The Spears Research Institute, HealthCare Chaplaincy, 307 East 60th Street, New York, NY, 10022, USA; 5 Department of Psychology, Marymount Manhattan College, 221 East 71st Street, New York, NY, 10021, USA; 6 Mount Sinai School of Medicine, Annenberg Building Floor 10, 1468 Madison Avenue, New York, NY, 10029, USA

**Keywords:** Chaplaincy care, Pastoral care, Health outcomes, End-of-life care, Hospice

## Abstract

**Background:**

Medicine has long acknowledged the role of chaplains in healthcare, but there is little research on the relationship between chaplaincy care and health outcomes. The present study examines the association between chaplaincy services and end-of-life care service choices.

**Methods:**

HealthCare Chaplaincy purchased the AHA survey database from the American Hospital Association. The Dartmouth Atlas of Health Care database was provided to HealthCare Chaplaincy by The Dartmouth Institute for Health Policy & Clinical Practice, with the permission of Dartmouth Atlas Co-Principal Investigator Elliot S. Fisher, M.D., M.P.H. The Dartmouth Atlas of Health Care is available interactively on-line at http://www.dartmouthatlas.org/. Patient data are aggregated at the hospital level in the Dartmouth Atlas of Health Care. IRB approval was not sought for the project because the data are available to the public through one means or another, and neither database contains data about individual patients, i.e. all the variables are measures of hospital characteristics. We combined and analyzed data from the American Hospital Association’s Annual Survey and outcome data from The Dartmouth Atlas of Health Care in a cross-sectional study of 3,585 hospitals. Two outcomes were examined: the percent of patients who (1) died in the hospital, and (2) were enrolled in hospice. Ordinary least squares regression was used to measure the association between the provision of chaplaincy services and each of the outcomes, controlling for six factors associated with hospital death rates.

**Results and discussion:**

The analyses found significantly lower rates of hospital deaths (β = .04, *p* < .05) and higher rates of hospice enrollment (β = .06, *p* < .001) for patients cared for in hospitals that provided chaplaincy services compared to hospitals that did not.

**Conclusions:**

The findings suggest that chaplaincy services may play a role in increasing hospice enrollment. This may be attributable to chaplains’ assistance to patients and families in making decisions about care at the end-of-life, perhaps by aligning their values and wishes with actual treatment plans. Additional research is warranted.

## Introduction

Most individuals in the United States (U.S.) who are seriously ill say they want to die at home [1]. Yet, research has consistently shown that the majority of older U.S. adults [2-4], especially those with chronic diseases [2,3,5], die in the hospital. This is the case in other countries, too, according to studies conducted in the 1990’s [6,7].

More recent research has found that high rates of hospital deaths among older adults and those with chronic illnesses exists in most of the countries in which it has been studied, including Australia [8], Belgium [9-11], Canada [12], Korea [13], Singapore [14], the U.K. [11], and the U.S. [15]. Exceptions to this observation are The Netherlands [11] and Sweden [16].

A number of factors have been examined that contribute to the high rates of hospital deaths. U.S. studies consistently have found that hospital deaths are related to hospital bed availability and vary across states and regions of the country, with hospital death rates being lowest in the Western states [3-5]. This association between hospital bed availability and rate of hospital deaths has been found in Belgium and the United Kingdom, as well [11].

Another geographically related variable that is associated with rates of hospital deaths is population density. Research in Belgium [10], Germany [17], and Taiwan [18] has shown that people living in urban areas are more likely to die in the hospital than those living in rural areas, although findings from other studies are inconsistent [2,7]. The observed relationship between hospital deaths and population density, as well as the regional differences, may be partly attributable to the number of available hospital beds per capita, with areas that have a greater number of hospital beds per person having higher rates of hospital deaths [3,19].

Geographically based (e.g. census tracks) measures of socio-economic status have also been found to be associated with hospital death rates. For example, Belgian [9] and American researchers [19] have reported that individuals from poorer neighborhoods have higher rates of hospital deaths than those from wealthier neighborhoods.

Recent studies have begun to explore the degree to which different healthcare services are associated with different end-of-life outcomes. For instance, an Australian study reported that community nursing services, which included emotional support for patients and families, were associated with lower hospital death rates [20]. Other Australian studies reported that community-based palliative care services were associated with lower rates of hospital deaths [8,21]. The lower rates of hospital deaths were associated with higher rates of institutional hospice deaths in one study [21] and higher rates of home deaths in the other [8]. A Belgian study also found that community-based palliative care services were associated with lower rates of hospital deaths and higher rates of home deaths [22].

U.S. research suggests that hospital-based palliative care programs can affect hospice enrollment and hospital deaths. One study, for example, reported that inpatient palliative care produced higher rates of referrals to home-based hospice programs than usual acute care [23], and another reported that palliative care teams successfully diverted patients into inpatient and home-based hospice programs [24]. In a randomized control trial, patients who received palliative care services had fewer admissions to intensive care units (ICU) and longer hospice stays [25]. Other research further indicates that palliative care teams may help to reduce hospital deaths, especially ICU deaths [26].

Chaplains have long been considered to be an integral part of the palliative care team [27] and they have routinely provided emotional and spiritual support to patients and family members even when palliative care services were not available [28-32]. However, only two studies to date have examined the effectiveness of chaplaincy care, one of which found chaplaincy care reduced patient anxiety [33] and the other of which reported that it helped patients contend with spiritual distress [34].

Although no previous research has examined the relationship between chaplaincy care and end-of-life outcomes, there is reason to believe that spiritual care, like that provided by chaplains, may affect end-of-life decision-making. Recent research has found that patients who say their spiritual needs were met by hospital staff were more likely to receive hospice care and less likely to receive aggressive treatment [35,36].

We have noticed that one of the things that chaplains do for patients is help them understand and feel comfortable with their core values and how these can be synthetic with their health care goals. Since people generally prefer to die at home, chaplains may help patients and families to resolve issues about end of life care, so that patients are less likely to die in the hospital.

### Study aims

The present study was designed to test two hypotheses about the provision of chaplaincy services and end-of-life outcomes. First, hospitals that provide chaplaincy services will have lower rates of hospital deaths than hospitals that do not provide chaplaincy services. Second, hospitals that provide chaplaincy services will have higher rates of hospice enrollment than hospitals that do not provide chaplaincy services.

## Methods

### Study sample

The study combined datasets from the American Hospital Association (AHA) and The Dartmouth Atlas of Health Care. The AHA regularly conducts surveys of its member hospitals in which hospital administrators are asked to report the facilities and services the hospital provides, along with various other characteristics of the hospital. The present study used data from the 2005 fiscal-year survey. The Dartmouth Atlas of Health Care combined data from the Center for Medicare and Medicaid Services on traditional, fee-for-service medical claims to track patients who died between January 1, 2001 and December 31, 2005, and had one or more of nine chronic diseases: congestive heart failure, chronic lung disease, cancer, coronary artery disease, renal failure, peripheral vascular disease, diabetes, chronic liver disease, and dementia. The Dartmouth Atlas uses discharge claims to assign patients to the hospital in which they died, or were admitted most often during the last two years of life. Patients who only had surgical admissions were excluded from The Dartmouth Atlas database.

Of the 4,271 healthcare facilities in The Dartmouth Atlas, 4,180 (97.9 %) were matched by name to hospitals in the AHA database. Of these, 3,603 completed the AHA annual survey for fiscal-year 2005. After excluding children’s, orthopedic, and obstetrics/gynecology hospitals, and rehabilitation and long-term care facilities, the sample consisted of 3,585 hospitals: 3,566 general medical, one surgical, eight heart, and ten cancer hospitals.

### Dependent variables

Two measures from The Dartmouth Atlas served as dependent variables: (1) the percent of patients who died in each hospital; and (2) the percent of patients who enrolled in home-based hospice.

### Independent variable

The independent variable for the study was whether or not a hospital provided chaplaincy services by hospital staff or through a vendor. The AHA Survey’s definition for “Chaplaincy/Pastoral Care Services” is: **“**A service ministering religious activities and providing pastoral counseling to patients, their families, and staff of a health care organization.” Hospitals that had chaplaincy services were coded as 1 and those that did not were coded as 0.

### Control variables

Six control variables were used in the analyses: (1) whether the hospital was located in a western state (Alaska, Arizona, California, Colorado, Hawaii, Idaho, Montana, Nevada, New Mexico, Oregon Utah, Washington, and Wyoming); (2) the population density of its catchment area; (3) number of hospital beds; (4) the proportion of Medicaid patients; (5) whether the hospital has a palliative care team; and (6) type of hospital. The three categorical variables (1, 5 and 6) were dummy coded as explained below.

It was essential to control for the region of the U.S. in which the hospital was located (i.e., western states), since several studies have found that hospital deaths are lower in the western states than in other states. [3-5]. This variable also controls for regional differences in palliative care and chaplaincy services [37-39]. Population density was included as a control variable in the analyses because it also has been found to be related to the rate of hospital deaths [17,18,22]. The AHA survey measures population density on a 0-6 scale, reflecting the size of the Metropolitan Statistical Area in which a hospital is located.

The next control variable, hospital beds, was included to control for bed availability, which is associated with hospital death rates [3,11,19], and the fact that larger hospitals (as measured by beds) are more likely to provide palliative and chaplaincy services [37-40]. This variable was measured by the AHA bed size code, which uses a 1-8 scale.

The proportion of each hospital’s Medicaid patients was used as a proxy for socio-economic status of patients [41]. It was calculated by dividing the hospital’s number of Medicaid discharges by its number of licensed beds. To control for the net effects of palliative care services, hospitals that had a palliative care program were dummy coded as 1 and those that did not have one were coded as 0. Similarly, we controlled for type of hospital by dummy coding the 19 specialty hospitals as 1 and the general hospitals as 0.

### Regression models

The association between chaplaincy and each of the dependent variables was tested by using ordinary least-squares (OLS) regression, controlling for western states, population density, number of hospitals beds, socio-economic status, type of hospital, and whether a hospital had a palliative care program. Because 95 hospitals did not have data on hospice enrollment, only 3,490 hospitals were used in the analysis of this dependent variable. Other analyses are described in the results section.

## Results

Table 1 presents the univariate statistics for the independent, dependent and control variables. Of the 3,585 hospitals in the sample, nearly 2,457 hospitals provided chaplaincy services (68.5 %), and 1,092 hospitals provided palliative care services (30.5 %). Just under 18 % were located in the 13 western states. The average rates of hospital deaths and hospice enrollment were approximately, 35 % and 29 %, respectively.

**Table 1:** Univariate Statistics for Independent, Dependent, and Control Variables

**(N = 3,585 Hospitals)**	**Number**	**Percent**
Hospitals with Chaplaincy Services	2457	68.5
Hospitals in Western States	630	17.6
Hospitals with Palliative Care	1092	30.5
Specialty Hospitals	19	0.6
	**Mean**	**S.D.**
Rate of Hospital Deaths	34.9	7.6
Rate of Hospice Enrollment	29.2	12.2
Population Density	2.2	2.4
Number of Hospital Beds	3.9	1.8
Socio-Economic Status (Proportion of Medicaid Patients)	1.5	2.9

On a scale of 0-6, the mean population density of 2.2 indicates that the average hospital in the sample was located in a geographical area with a population of over 250,000 residents. The average size of hospitals in the study was between 100 and 200 beds, based on the AHA bed size code.

Table 2 presents the results of the OLS regression models predicting rates of hospital deaths and hospice enrollment. The values given in the table are standardized beta’s (β’s). which show the unique association of chaplaincy services and each of the control variables with the two dependent variables: hospital deaths and hospice enrollment.

**Table 2:** Estimated Net Association of Chaplaincy Services Variables with the Rate of Hospital Deaths and Rate of Hospice Enrollment, Controlling for the Six other Variables in the Model

**Variables**	**Hospital Deaths**	**Hospice Enrollment**
Western States	-0.12***	0.02
Population Density	0.02	0.29***
Number of Hospital Beds	0.27***	0.06**
Socio-Economic Status	0.02	-0.08***
Palliative Care	-0.07***	-0.02
Specialty Hospitals	0.03	0.01
Chaplaincy Services	-0.04*	0.06***
Adjusted *R*^2^	0.08	0.12

* *p* < .05 ** *p* < .01 *** *p* < .001 Values in the table are standardized beta’s.

As hypothesized, chaplaincy services were associated with significantly lower rates of hospital deaths (β = -.04, *p* < .01) compared to hospitals that did not provide chaplaincy services, controlling for region of the country, population density, hospital beds, socio-economic status, palliative care and type of hospital (see Table 2). As expected, hospitals located in western states and those with palliative care teams had significantly lower rates of hospital deaths.

Number of hospital beds was positively and significantly related to hospital deaths, but population was not. However, these two variables were positively correlated, and the association between hospital deaths and population density (β = 0.14) was statistically significant (*p* < .001) when the model was re-run without number of beds. This supports previous findings that population density is positively related to hospital deaths and shows that the inclusion of number of beds in the model captured a substantial portion of the variation in hospital deaths that would otherwise have been attributable to population density.

As hypothesized, chaplaincy services also were associated with significantly higher rates of hospice enrollment (β = .06, *p* < .001). Population density and number of beds were positively related to hospice enrollment, while socio-economic status was negatively related to enrollment.

## Discussion

The findings support both hypotheses: that chaplaincy services are significantly associated with (1) lower rates of hospital deaths and (2) higher rates of hospice enrollment. Although these associations are relatively small, they can have profound consequences for patients and families. One would not expect appreciably larger net effects of specific services, since many unknown variables also contribute to the outcomes being examined. Nevertheless, the fact that the regression model for rates of hospital deaths replicated the effects of variables known to be related to hospital death rates from previous research lends credence to the generalizability of the findings. While palliative care exhibited the expected negative association with hospital deaths, it is not apparent why it did not have a significant positive association with hospice enrollment.

Chaplains often help patients and family members to feel comfortable with their core values and to see how these can be congruent with their health care goals. Since most people prefer to die at home [1,42,43], discussions of values and goals with chaplains might partially account for the observed association of chaplaincy services with decreased hospital deaths and increased hospice enrollment.

The present results are consistent with recent research that patients who said their spiritual needs were met by hospital staff were more likely to receive hospice care and less likely to receive aggressive treatment [35,36]. Related research has found that families of dying patients who felt supported [44], and were able to discuss spiritual issues when making end-of-life decisions were more satisfied with those decisions [44].

Other research has found that meeting the spiritual needs of patients is associated with higher levels of patient [45] and family [46,47] satisfaction with the quality of overall care. Meeting spiritual needs is particularly important at the end of life in order to achieve spiritual and psychological healing when physical healing is no longer possible, and palliation is the goal [48,49]. At this time of life, it is essential to align treatment with the patient’s goals [50], which are often influenced by spirituality and religion [51].

A recent study of patients in a palliative care unit with end-stage cancer found that 88 % of patients expressed the desire to work with a chaplain [52]. Roughly half of the patients indicated they would like the chaplain to provide a sense of “presence,” listen to them, visit with them, or accompany them on their journey. The more religious patients also desired various spiritual interventions from the hospital chaplain.

Surveys of hospital administrators in the U.S. have found that professional chaplains are expected to play a number of roles [53,54]. In addition to providing spiritual and emotional support to patients and families, generally, chaplains are also expected to help patients and family members address end-of-life issues and deal with difficult decisions. Related U.S. research indicates that hospital staff refer patients and families to chaplains for such reasons [32], and that chaplains spend a considerable amount of time dealing with these kinds of issues [55,56]. Moreover, Australian studies have found that the majority of hospital chaplains helped patients and families make treatment decisions [57], and that many chaplains assisted families and staff dealing with pain control and life support issues [58-60].

This study has a number of limitations, including the fact that the AHA survey only indicated whether or not a hospital provided chaplaincy services. It did not provide information about the specific nature, scope, and quality of the services. Another limitation is that the patient outcome data are aggregated across a 5-year period (2001-2005), and there is evidence that the provision of chaplaincy services increased somewhat during this time [37]. This introduces some degree of error to the models, which are based on the hospital services that existed in fiscal-year 2005 (2004-2005).

Although the analyses controlled for a number of variables that have been shown to be associated with end-of-life outcomes, the presence of chaplaincy services could still be a proxy for some other variable or variables. It could, for example, be an indication that a hospital is committed to providing patient-centered and family-focused care in which all members of the team are dedicated to listening to the patient and aligning care with patient and family wishes. Since the evidence indicates that this stance generally leads to less use of hospital resources at the end-of –life, these hospitals might have fewer hospital deaths even if they did not have chaplains. Nonetheless, this type of care, whether provided by a chaplain or another discipline, appears to have an impact.

Its limitations notwithstanding, we believe the present study provides support for the possible role of chaplains in reducing hospital deaths and increasing hospice enrollment. This could be attributable to the fact that chaplains assist patients and families in making decisions about care at the end-of-life and aligning their values and wishes with actual treatment plans.

## Conclusions

The findings suggest that chaplaincy services may play a role in decreasing hospital deaths and increasing hospice enrollment. This may be attributable to chaplains’ assistance to patients and families in making decisions about care at the end-of-life, perhaps by aligning their values and wishes with actual treatment plans. Additional research is warranted.

## Competing interests

The authors declare that they have no competing interests.

## Authors’ contributions

KJF designed the study with KG, NRS and MC, all of whom wrote the initial draft. KJF, LLE and GFH were the primary authors. KJF conducted the statistical analyses. All the authors reviewed drafts and approved the final content.

## Pre-publication history

The pre-publication history for this paper can be accessed here:

http://www.biomedcentral.com/1472-684X/11/10/prepub
